# Supplementary material for: Comparative multiomics analysis of cell physiological state after culture in a basket bioreactor
Source: Sci Rep. 2022 Nov 23;12:20161. doi: 10.1038/s41598-022-24687-4 (PMC9686226; doi:10.1038/s41598-022-24687-4)
Supplement: Supplementary file 1 — Supplementary Information 1. [file 41598_2022_24687_MOESM1_ESM.zip › raw data/Transcriptomic raw data/6.Enrichment/2.KEGG/TE1vsTC1/DOWN/plot/TE1vsTC1.html]

### TE1vsTC1 KEGG通路富集列表

---

| KEGGID | Description | GeneRatio | BgRatio | pvalue | padj | geneID | geneName | keggID | Count |
| --- | --- | --- | --- | --- | --- | --- | --- | --- | --- |
| csab04621 | NOD-like receptor signaling pathway | 10/88 | 131/5900 | 0.0000214 | 0.0047134 | 103224057/103241278/103224476/103239154/103230198/103235789/103224480/103215179/103242710/103239156 | LOC103224057/IL1B/LOC103224476/OAS1/IKBKE/CXCL1/LOC103224480/CARD6/CCL2/OAS2 | csab:103224057/csab:103241278/csab:103224476/csab:103239154/csab:103230198/csab:103235798/csab:103224480/csab:103215179/csab:103242710/csab:103239156 | 10 |
| csab04060 | Cytokine-cytokine receptor interaction | 10/88 | 170/5900 | 0.0001960 | 0.0215594 | 103241278/103237005/103219268/103235789/103225587/103242551/103242198/103225819/103223920/103242710 | IL1B/IL7/CNTFR/CXCL1/TNFRSF1B/TNFRSF6B/CXCL16/TNFRSF18/IL6R/CCL2 | csab:103241278/csab:103237005/csab:103219268/csab:103235798/csab:103225587/csab:103242551/csab:103242198/csab:103225819/csab:103223920/csab:103242710 | 10 |
| csab05171 | Coronavirus disease - COVID-19 | 9/88 | 163/5900 | 0.0006621 | 0.0485523 | 103241278/103221732/103239154/103230198/103224900/103233571/103223920/103242710/103239156 | IL1B/LOC103221732/OAS1/IKBKE/PIK3R3/CFD/IL6R/CCL2/OAS2 | csab:103241278/csab:103221732/csab:103239154/csab:103230198/csab:103224900/csab:103233571/csab:103223920/csab:103242710/csab:103239156 | 9 |
| csab04610 | Complement and coagulation cascades | 5/88 | 55/5900 | 0.0012739 | 0.0700660 | 103229609/103231401/103215685/103221732/103233571 | SERPINA1/ITGAX/PLAT/LOC103221732/CFD | csab:103229609/csab:103231401/csab:103215685/csab:103221732/csab:103233571 | 5 |
| csab05164 | Influenza A | 7/88 | 116/5900 | 0.0016161 | 0.0711097 | 103241278/103239154/103230198/103224900/103232396/103242710/103239156 | IL1B/OAS1/IKBKE/PIK3R3/NXF3/CCL2/OAS2 | csab:103241278/csab:103239154/csab:103230198/csab:103224900/csab:103232396/csab:103242710/csab:103239156 | 7 |
| csab05417 | Lipid and atherosclerosis | 8/88 | 160/5900 | 0.0025049 | 0.0918454 | 103241278/103240818/103230198/103235789/103223257/103224900/103223677/103242710 | IL1B/SOD2/IKBKE/CXCL1/NCF4/PIK3R3/HSPA6/CCL2 | csab:103241278/csab:103240818/csab:103230198/csab:103235798/csab:103223257/csab:103224900/csab:103223677/csab:103242710 | 8 |
| csab04668 | TNF signaling pathway | 6/88 | 98/5900 | 0.0032582 | 0.1006492 | 103241278/103235789/103225587/103218917/103224900/103242710 | IL1B/CXCL1/TNFRSF1B/TRAF1/PIK3R3/CCL2 | csab:103241278/csab:103235798/csab:103225587/csab:103218917/csab:103224900/csab:103242710 | 6 |
| csab00590 | Arachidonic acid metabolism | 4/88 | 43/5900 | 0.0036600 | 0.1006492 | 103235528/103245108/103218549/103220573 | PLA2G4C/LTC4S/CBR3/PLB1 | csab:103235528/csab:103245108/csab:103218549/csab:103220573 | 4 |
| csab05162 | Measles | 6/88 | 110/5900 | 0.0057524 | 0.1262261 | 103241278/103239154/103230198/103224900/103223677/103239156 | IL1B/OAS1/IKBKE/PIK3R3/HSPA6/OAS2 | csab:103241278/csab:103239154/csab:103230198/csab:103224900/csab:103223677/csab:103239156 | 6 |
| csab05418 | Fluid shear stress and atherosclerosis | 6/88 | 111/5900 | 0.0060096 | 0.1262261 | 103241278/103215685/103220724/103218213/103224900/103242710 | IL1B/PLAT/SDC1/GPC1/PIK3R3/CCL2 | csab:103241278/csab:103215685/csab:103220724/csab:103218213/csab:103224900/csab:103242710 | 6 |
| csab04061 | Viral protein interaction with cytokine and cytokine receptor | 4/88 | 50/5900 | 0.0063113 | 0.1262261 | 103235789/103225587/103223920/103242710 | CXCL1/TNFRSF1B/IL6R/CCL2 | csab:103235798/csab:103225587/csab:103223920/csab:103242710 | 4 |
| csab05144 | Malaria | 3/88 | 35/5900 | 0.0148753 | 0.2717126 | 103241278/103220724/103242710 | IL1B/SDC1/CCL2 | csab:103241278/csab:103220724/csab:103242710 | 3 |
| csab00565 | Ether lipid metabolism | 3/88 | 36/5900 | 0.0160557 | 0.2717126 | 103235528/103224742/103220573 | PLA2G4C/PLPP3/PLB1 | csab:103235528/csab:103224742/csab:103220573 | 3 |
| csab04657 | IL-17 signaling pathway | 4/88 | 69/5900 | 0.0191426 | 0.2986148 | 103241278/103230198/103235789/103242710 | IL1B/IKBKE/CXCL1/CCL2 | csab:103241278/csab:103230198/csab:103235798/csab:103242710 | 4 |
| csab00592 | alpha-Linolenic acid metabolism | 2/88 | 15/5900 | 0.0203601 | 0.2986148 | 103235528/103220573 | PLA2G4C/PLB1 | csab:103235528/csab:103220573 | 2 |
| csab00591 | Linoleic acid metabolism | 2/88 | 16/5900 | 0.0230458 | 0.3168796 | 103235528/103220573 | PLA2G4C/PLB1 | csab:103235528/csab:103220573 | 2 |
| csab04630 | JAK-STAT signaling pathway | 5/88 | 111/5900 | 0.0245342 | 0.3175013 | 103237005/103218491/103219268/103224900/103223920 | IL7/PTPN6/CNTFR/PIK3R3/IL6R | csab:103237005/csab:103218491/csab:103219268/csab:103224900/csab:103223920 | 5 |
| csab05134 | Legionellosis | 3/88 | 44/5900 | 0.0273568 | 0.3343605 | 103241278/103235789/103223677 | IL1B/CXCL1/HSPA6 | csab:103241278/csab:103235798/csab:103223677 | 3 |
| csab00564 | Glycerophospholipid metabolism | 4/88 | 80/5900 | 0.0309800 | 0.3587155 | 103235528/103228224/103224742/103220573 | PLA2G4C/PLA1A/PLPP3/PLB1 | csab:103235528/csab:103228224/csab:103224742/csab:103220573 | 4 |
| csab04066 | HIF-1 signaling pathway | 4/88 | 83/5900 | 0.0348155 | 0.3678703 | 103242625/103218486/103224900/103223920 | ALDOC/ENO2/PIK3R3/IL6R | csab:103242625/csab:103218486/csab:103224900/csab:103223920 | 4 |
| csab00770 | Pantothenate and CoA biosynthesis | 2/88 | 20/5900 | 0.0351149 | 0.3678703 | 103240354/103240351 | LOC103240354/VNN2 | csab:103240354/csab:103240351 | 2 |
| csab04940 | Type I diabetes mellitus | 2/88 | 21/5900 | 0.0384416 | 0.3800826 | 103241278/103217907 | IL1B/PTPRN | csab:103241278/csab:103217907 | 2 |
| csab05205 | Proteoglycans in cancer | 6/88 | 169/5900 | 0.0397359 | 0.3800826 | 103223483/103218491/103220724/103224207/103218213/103224900 | WNT7B/PTPN6/SDC1/WNT2B/GPC1/PIK3R3 | csab:103223483/csab:103218491/csab:103220724/csab:103224207/csab:103218213/csab:103224900 | 6 |
| csab04213 | Longevity regulating pathway - multiple species | 3/88 | 53/5900 | 0.0440043 | 0.4033724 | 103240818/103224900/103223677 | SOD2/PIK3R3/HSPA6 | csab:103240818/csab:103224900/csab:103223677 | 3 |
| csab05140 | Leishmaniasis | 3/88 | 54/5900 | 0.0461054 | 0.4057274 | 103241278/103218491/103223257 | IL1B/PTPN6/NCF4 | csab:103241278/csab:103218491/csab:103223257 | 3 |
| csab04640 | Hematopoietic cell lineage | 3/88 | 56/5900 | 0.0504545 | 0.4269226 | 103241278/103237005/103223920 | IL1B/IL7/IL6R | csab:103241278/csab:103237005/csab:103223920 | 3 |
| csab04014 | Ras signaling pathway | 6/88 | 184/5900 | 0.0559966 | 0.4499432 | 103230197/103246479/103235528/103228224/103224900/103223351 | RASSF5/FGFR3/PLA2G4C/PLA1A/PIK3R3/RIN1 | csab:103230197/csab:103246479/csab:103235528/csab:103228224/csab:103224900/csab:103223351 | 6 |
| csab04721 | Synaptic vesicle cycle | 3/88 | 60/5900 | 0.0597284 | 0.4499432 | 103246629/103246502/103239806 | STX1A/SNAP25/DNM1 | csab:103246629/csab:103246502/csab:103239806 | 3 |
| csab04960 | Aldosterone-regulated sodium reabsorption | 2/88 | 27/5900 | 0.0606816 | 0.4499432 | 103224900/103229709 | PIK3R3/SCNN1B | csab:103224900/csab:103229709 | 2 |
| csab05323 | Rheumatoid arthritis | 3/88 | 61/5900 | 0.0621641 | 0.4499432 | 103241278/103235789/103242710 | IL1B/CXCL1/CCL2 | csab:103241278/csab:103235798/csab:103242710 | 3 |
| csab04936 | Alcoholic liver disease | 4/88 | 101/5900 | 0.0634011 | 0.4499432 | 103241278/103221732/103230198/103235789 | IL1B/LOC103221732/IKBKE/CXCL1 | csab:103241278/csab:103221732/csab:103230198/csab:103235798 | 4 |
| csab04911 | Insulin secretion | 3/88 | 64/5900 | 0.0697446 | 0.4794938 | 103226636/103246629/103246502 | PCLO/STX1A/SNAP25 | csab:103226636/csab:103246629/csab:103246502 | 3 |
| csab01521 | EGFR tyrosine kinase inhibitor resistance | 3/88 | 69/5900 | 0.0832568 | 0.5466501 | 103246479/103224900/103223920 | FGFR3/PIK3R3/IL6R | csab:103246479/csab:103224900/csab:103223920 | 3 |
| csab05169 | Epstein-Barr virus infection | 5/88 | 157/5900 | 0.0844823 | 0.5466501 | 103239154/103230198/103224900/103221664/103239156 | OAS1/IKBKE/PIK3R3/LOC103221664/OAS2 | csab:103239154/csab:103230198/csab:103224900/csab:103221664/csab:103239156 | 5 |
| csab04550 | Signaling pathways regulating pluripotency of stem cells | 4/88 | 116/5900 | 0.0943351 | 0.5536671 | 103223483/103246479/103224207/103224900 | WNT7B/FGFR3/WNT2B/PIK3R3 | csab:103223483/csab:103246479/csab:103224207/csab:103224900 | 4 |
| csab00830 | Retinol metabolism | 2/88 | 35/5900 | 0.0953759 | 0.5536671 | 103236970/103225584 | RDH10/DHRS3 | csab:103236970/csab:103225584 | 2 |
| csab04620 | Toll-like receptor signaling pathway | 3/88 | 75/5900 | 0.1008290 | 0.5536671 | 103241278/103230198/103224900 | IL1B/IKBKE/PIK3R3 | csab:103241278/csab:103230198/csab:103224900 | 3 |
| csab05146 | Amoebiasis | 3/88 | 75/5900 | 0.1008290 | 0.5536671 | 103241278/103235789/103224900 | IL1B/CXCL1/PIK3R3 | csab:103241278/csab:103235798/csab:103224900 | 3 |
| csab05200 | Pathways in cancer | 10/88 | 428/5900 | 0.1031101 | 0.5536671 | 103220342/103237005/103230197/103223483/103246479/103224207/103218917/103224900/103239621/103223920 | EPAS1/IL7/RASSF5/WNT7B/FGFR3/WNT2B/TRAF1/PIK3R3/NOTCH1/IL6R | csab:103220342/csab:103237005/csab:103230197/csab:103223483/csab:103246479/csab:103224207/csab:103218917/csab:103224900/csab:103239621/csab:103223920 | 10 |
| csab04666 | Fc gamma R-mediated phagocytosis | 3/88 | 77/5900 | 0.1069936 | 0.5536671 | 103235528/103224742/103224900 | PLA2G4C/PLPP3/PIK3R3 | csab:103235528/csab:103224742/csab:103224900 | 3 |
| csab04750 | Inflammatory mediator regulation of TRP channels | 3/88 | 78/5900 | 0.1101306 | 0.5536671 | 103241278/103235528/103224900 | IL1B/PLA2G4C/PIK3R3 | csab:103241278/csab:103235528/csab:103224900 | 3 |
| csab04064 | NF-kappa B signaling pathway | 3/88 | 79/5900 | 0.1133031 | 0.5536671 | 103241278/103235789/103218917 | IL1B/CXCL1/TRAF1 | csab:103241278/csab:103235798/csab:103218917 | 3 |
| csab04623 | Cytosolic DNA-sensing pathway | 2/88 | 39/5900 | 0.1144056 | 0.5536671 | 103241278/103230198 | IL1B/IKBKE | csab:103241278/csab:103230198 | 2 |
| csab05163 | Human cytomegalovirus infection | 5/88 | 173/5900 | 0.1153096 | 0.5536671 | 103241278/103224900/103221664/103223920/103242710 | IL1B/PIK3R3/LOC103221664/IL6R/CCL2 | csab:103241278/csab:103224900/csab:103221664/csab:103223920/csab:103242710 | 5 |
| csab05160 | Hepatitis C | 4/88 | 125/5900 | 0.1157668 | 0.5536671 | 103239154/103230198/103224900/103239156 | OAS1/IKBKE/PIK3R3/OAS2 | csab:103239154/csab:103230198/csab:103224900/csab:103239156 | 4 |
| csab05224 | Breast cancer | 4/88 | 125/5900 | 0.1157668 | 0.5536671 | 103223483/103224207/103224900/103239621 | WNT7B/WNT2B/PIK3R3/NOTCH1 | csab:103223483/csab:103224207/csab:103224900/csab:103239621 | 4 |
| csab04072 | Phospholipase D signaling pathway | 4/88 | 128/5900 | 0.1233523 | 0.5664193 | 103235528/103224742/103224900/103239806 | PLA2G4C/PLPP3/PIK3R3/DNM1 | csab:103235528/csab:103224742/csab:103224900/csab:103239806 | 4 |
| csab04625 | C-type lectin receptor signaling pathway | 3/88 | 83/5900 | 0.1263351 | 0.5664193 | 103241278/103230198/103224900 | IL1B/IKBKE/PIK3R3 | csab:103241278/csab:103230198/csab:103224900 | 3 |
| csab05231 | Choline metabolism in cancer | 3/88 | 85/5900 | 0.1330468 | 0.5664193 | 103235528/103224742/103224900 | PLA2G4C/PLPP3/PIK3R3 | csab:103235528/csab:103224742/csab:103224900 | 3 |
| csab04150 | mTOR signaling pathway | 4/88 | 132/5900 | 0.1337902 | 0.5664193 | 103223483/103224207/103246611/103224900 | WNT7B/WNT2B/CASTOR2/PIK3R3 | csab:103223483/csab:103224207/csab:103246611/csab:103224900 | 4 |
| csab04612 | Antigen processing and presentation | 2/88 | 43/5900 | 0.1343095 | 0.5664193 | 103223677/103221664 | HSPA6/LOC103221664 | csab:103223677/csab:103221664 | 2 |
| csab05142 | Chagas disease | 3/88 | 86/5900 | 0.1364491 | 0.5664193 | 103241278/103224900/103242710 | IL1B/PIK3R3/CCL2 | csab:103241278/csab:103224900/csab:103242710 | 3 |
| csab04062 | Chemokine signaling pathway | 4/88 | 133/5900 | 0.1364556 | 0.5664193 | 103235789/103242198/103224900/103242710 | CXCL1/CXCL16/PIK3R3/CCL2 | csab:103235798/csab:103242198/csab:103224900/csab:103242710 | 4 |
| csab00600 | Sphingolipid metabolism | 2/88 | 44/5900 | 0.1394025 | 0.5679360 | 103224742/103233234 | PLPP3/SMPD3 | csab:103224742/csab:103233234 | 2 |
| csab00980 | Metabolism of xenobiotics by cytochrome P450 | 2/88 | 45/5900 | 0.1445374 | 0.5781495 | 103230154/103218549 | HSD11B1/CBR3 | csab:103230154/csab:103218549 | 2 |
| csab00430 | Taurine and hypotaurine metabolism | 1/88 | 11/5900 | 0.1524835 | 0.5911582 | 103230592 | FMO4 | csab:103230592 | 1 |
| csab05150 | Staphylococcus aureus infection | 2/88 | 47/5900 | 0.1549236 | 0.5911582 | 103221732/103233571 | LOC103221732/CFD | csab:103221732/csab:103233571 | 2 |
| csab05168 | Herpes simplex virus 1 infection | 9/88 | 410/5900 | 0.1559076 | 0.5911582 | 103241278/103239154/103230198/103224900/103232396/103221664/103219115/103242710/103239156 | IL1B/OAS1/IKBKE/PIK3R3/NXF3/LOC103221664/ZNF189/CCL2/OAS2 | csab:103241278/csab:103239154/csab:103230198/csab:103224900/csab:103232396/csab:103221664/csab:103219115/csab:103242710/csab:103239156 | 9 |
| csab04310 | Wnt signaling pathway | 4/88 | 141/5900 | 0.1585379 | 0.5911582 | 103223483/103215672/103224207/103237471 | WNT7B/SFRP1/WNT2B/CCN4 | csab:103223483/csab:103215672/csab:103224207/csab:103237471 | 4 |
| csab04370 | VEGF signaling pathway | 2/88 | 49/5900 | 0.1654501 | 0.6066502 | 103235528/103224900 | PLA2G4C/PIK3R3 | csab:103235528/csab:103224900 | 2 |
| csab04933 | AGE-RAGE signaling pathway in diabetic complications | 3/88 | 95/5900 | 0.1683409 | 0.6071309 | 103241278/103224900/103242710 | IL1B/PIK3R3/CCL2 | csab:103241278/csab:103224900/csab:103242710 | 3 |
| csab04270 | Vascular smooth muscle contraction | 3/88 | 99/5900 | 0.1831658 | 0.6220491 | 103235528/103240389/103223064 | PLA2G4C/ADM/ADORA2A | csab:103235528/csab:103240389/csab:103223064 | 3 |
| csab00010 | Glycolysis / Gluconeogenesis | 2/88 | 53/5900 | 0.1868562 | 0.6220491 | 103242625/103218486 | ALDOC/ENO2 | csab:103242625/csab:103218486 | 2 |
| csab05133 | Pertussis | 2/88 | 53/5900 | 0.1868562 | 0.6220491 | 103241278/103221732 | IL1B/LOC103221732 | csab:103241278/csab:103221732 | 2 |
| csab04977 | Vitamin digestion and absorption | 1/88 | 14/5900 | 0.1899185 | 0.6220491 | 103220573 | PLB1 | csab:103220573 | 1 |
| csab04380 | Osteoclast differentiation | 3/88 | 101/5900 | 0.1907081 | 0.6220491 | 103241278/103223257/103224900 | IL1B/NCF4/PIK3R3 | csab:103241278/csab:103223257/csab:103224900 | 3 |
| csab04670 | Leukocyte transendothelial migration | 3/88 | 101/5900 | 0.1907081 | 0.6220491 | 103230197/103223257/103224900 | RASSF5/NCF4/PIK3R3 | csab:103230197/csab:103223257/csab:103224900 | 3 |
| csab04664 | Fc epsilon RI signaling pathway | 2/88 | 54/5900 | 0.1922697 | 0.6220491 | 103235528/103224900 | PLA2G4C/PIK3R3 | csab:103235528/csab:103224900 | 2 |
| csab05217 | Basal cell carcinoma | 2/88 | 56/5900 | 0.2031579 | 0.6287450 | 103223483/103224207 | WNT7B/WNT2B | csab:103223483/csab:103224207 | 2 |
| csab04919 | Thyroid hormone signaling pathway | 3/88 | 105/5900 | 0.2060256 | 0.6287450 | 103232196/103224900/103239621 | SLC16A2/PIK3R3/NOTCH1 | csab:103232196/csab:103224900/csab:103239621 | 3 |
| csab05020 | Prion disease | 5/88 | 211/5900 | 0.2060685 | 0.6287450 | 103241278/103223257/103224900/103239621/103223677 | IL1B/NCF4/PIK3R3/NOTCH1/HSPA6 | csab:103241278/csab:103223257/csab:103224900/csab:103239621/csab:103223677 | 5 |
| csab01230 | Biosynthesis of amino acids | 2/88 | 57/5900 | 0.2086290 | 0.6287450 | 103242625/103218486 | ALDOC/ENO2 | csab:103242625/csab:103218486 | 2 |
| csab04662 | B cell receptor signaling pathway | 2/88 | 57/5900 | 0.2086290 | 0.6287450 | 103218491/103224900 | PTPN6/PIK3R3 | csab:103218491/csab:103224900 | 2 |
| csab04210 | Apoptosis | 3/88 | 107/5900 | 0.2137902 | 0.6355926 | 103218917/103245241/103224900 | TRAF1/CTSH/PIK3R3 | csab:103218917/csab:103245241/csab:103224900 | 3 |
| csab05230 | Central carbon metabolism in cancer | 2/88 | 60/5900 | 0.2251302 | 0.6603821 | 103246479/103224900 | FGFR3/PIK3R3 | csab:103246479/csab:103224900 | 2 |
| csab05211 | Renal cell carcinoma | 2/88 | 61/5900 | 0.2306545 | 0.6670561 | 103220342/103224900 | EPAS1/PIK3R3 | csab:103220342/csab:103224900 | 2 |
| csab05135 | Yersinia infection | 3/88 | 112/5900 | 0.2334696 | 0.6670561 | 103241278/103224900/103242710 | IL1B/PIK3R3/CCL2 | csab:103241278/csab:103224900/csab:103242710 | 3 |
| csab05332 | Graft-versus-host disease | 1/88 | 18/5900 | 0.2372999 | 0.6693073 | 103241278 | IL1B | csab:103241278 | 1 |
| csab05223 | Non-small cell lung cancer | 2/88 | 63/5900 | 0.2417296 | 0.6731711 | 103230197/103224900 | RASSF5/PIK3R3 | csab:103230197/csab:103224900 | 2 |
| csab05320 | Autoimmune thyroid disease | 1/88 | 19/5900 | 0.2487105 | 0.6822872 | 103237469 | TG | csab:103237469 | 1 |
| csab04068 | FoxO signaling pathway | 3/88 | 117/5900 | 0.2534693 | 0.6822872 | 103240818/103245773/103224900 | SOD2/SGK2/PIK3R3 | csab:103240818/csab:103245773/csab:103224900 | 3 |
| csab04015 | Rap1 signaling pathway | 4/88 | 172/5900 | 0.2543070 | 0.6822872 | 103230197/103246479/103223064/103224900 | RASSF5/FGFR3/ADORA2A/PIK3R3 | csab:103230197/csab:103246479/csab:103223064/csab:103224900 | 4 |
| csab05100 | Bacterial invasion of epithelial cells | 2/88 | 66/5900 | 0.2583870 | 0.6831541 | 103224900/103239806 | PIK3R3/DNM1 | csab:103224900/csab:103239806 | 2 |
| csab04142 | Lysosome | 3/88 | 119/5900 | 0.2615420 | 0.6831541 | 103242313/103245241/103243148 | CD68/CTSH/ARSG | csab:103242313/csab:103245241/csab:103243148 | 3 |
| csab04650 | Natural killer cell mediated cytotoxicity | 2/88 | 67/5900 | 0.2639459 | 0.6831541 | 103218491/103224900 | PTPN6/PIK3R3 | csab:103218491/csab:103224900 | 2 |
| csab00650 | Butanoate metabolism | 1/88 | 21/5900 | 0.2710280 | 0.6853580 | 103227954 | ABAT | csab:103227954 | 1 |
| csab05143 | African trypanosomiasis | 1/88 | 21/5900 | 0.2710280 | 0.6853580 | 103241278 | IL1B | csab:103241278 | 1 |
| csab00515 | Mannose type O-glycan biosynthesis | 1/88 | 22/5900 | 0.2819396 | 0.7025453 | 103242981 | MGAT5B | csab:103242981 | 1 |
| csab04810 | Regulation of actin cytoskeleton | 4/88 | 181/5900 | 0.2842115 | 0.7025453 | 103231401/103246479/103224900/103223856 | ITGAX/FGFR3/PIK3R3/IQGAP3 | csab:103231401/csab:103246479/csab:103224900/csab:103223856 | 4 |
| csab04975 | Fat digestion and absorption | 1/88 | 23/5900 | 0.2926897 | 0.7063790 | 103224742 | PLPP3 | csab:103224742 | 1 |
| csab05226 | Gastric cancer | 3/88 | 127/5900 | 0.2941337 | 0.7063790 | 103223483/103224207/103224900 | WNT7B/WNT2B/PIK3R3 | csab:103223483/csab:103224207/csab:103224900 | 3 |
| csab05235 | PD-L1 expression and PD-1 checkpoint pathway in cancer | 2/88 | 74/5900 | 0.3028191 | 0.7063790 | 103218491/103224900 | PTPN6/PIK3R3 | csab:103218491/csab:103224900 | 2 |
| csab00030 | Pentose phosphate pathway | 1/88 | 24/5900 | 0.3032807 | 0.7063790 | 103242625 | ALDOC | csab:103242625 | 1 |
| csab04010 | MAPK signaling pathway | 5/88 | 247/5900 | 0.3078243 | 0.7063790 | 103241278/103246479/103235528/103225290/103223677 | IL1B/FGFR3/PLA2G4C/MAP3K6/HSPA6 | csab:103241278/csab:103246479/csab:103235528/csab:103225290/csab:103223677 | 5 |
| csab01523 | Antifolate resistance | 1/88 | 25/5900 | 0.3137149 | 0.7063790 | 103241278 | IL1B | csab:103241278 | 1 |
| csab04211 | Longevity regulating pathway | 2/88 | 76/5900 | 0.3138790 | 0.7063790 | 103240818/103224900 | SOD2/PIK3R3 | csab:103240818/csab:103224900 | 2 |
| csab04916 | Melanogenesis | 2/88 | 76/5900 | 0.3138790 | 0.7063790 | 103223483/103224207 | WNT7B/WNT2B | csab:103223483/csab:103224207 | 2 |
| csab04932 | Non-alcoholic fatty liver disease | 3/88 | 132/5900 | 0.3146597 | 0.7063790 | 103241278/103224900/103223920 | IL1B/PIK3R3/IL6R | csab:103241278/csab:103224900/csab:103223920 | 3 |
| csab04660 | T cell receptor signaling pathway | 2/88 | 80/5900 | 0.3358833 | 0.7349696 | 103218491/103224900 | PTPN6/PIK3R3 | csab:103218491/csab:103224900 | 2 |
| csab04659 | Th17 cell differentiation | 2/88 | 81/5900 | 0.3413550 | 0.7349696 | 103241278/103223920 | IL1B/IL6R | csab:103241278/csab:103223920 | 2 |
| csab00250 | Alanine, aspartate and glutamate metabolism | 1/88 | 28/5900 | 0.3440994 | 0.7349696 | 103227954 | ABAT | csab:103227954 | 1 |
| csab00410 | beta-Alanine metabolism | 1/88 | 28/5900 | 0.3440994 | 0.7349696 | 103227954 | ABAT | csab:103227954 | 1 |
| csab00640 | Propanoate metabolism | 1/88 | 28/5900 | 0.3440994 | 0.7349696 | 103227954 | ABAT | csab:103227954 | 1 |
| csab05215 | Prostate cancer | 2/88 | 85/5900 | 0.3630989 | 0.7477490 | 103215685/103224900 | PLAT/PIK3R3 | csab:103215685/csab:103224900 | 2 |
| csab05206 | MicroRNAs in cancer | 3/88 | 145/5900 | 0.3680860 | 0.7477490 | 103246479/103224900/103239621 | FGFR3/PIK3R3/NOTCH1 | csab:103246479/csab:103224900/csab:103239621 | 3 |
| csab05225 | Hepatocellular carcinoma | 3/88 | 146/5900 | 0.3721792 | 0.7477490 | 103223483/103224207/103224900 | WNT7B/WNT2B/PIK3R3 | csab:103223483/csab:103224207/csab:103224900 | 3 |
| csab04672 | Intestinal immune network for IgA production | 1/88 | 31/5900 | 0.3731533 | 0.7477490 | 103230184 | PIGR | csab:103230184 | 1 |
| csab04973 | Carbohydrate digestion and absorption | 1/88 | 31/5900 | 0.3731533 | 0.7477490 | 103224900 | PIK3R3 | csab:103224900 | 1 |
| csab01522 | Endocrine resistance | 2/88 | 87/5900 | 0.3738745 | 0.7477490 | 103224900/103239621 | PIK3R3/NOTCH1 | csab:103224900/csab:103239621 | 2 |
| csab05222 | Small cell lung cancer | 2/88 | 87/5900 | 0.3738745 | 0.7477490 | 103218917/103224900 | TRAF1/PIK3R3 | csab:103218917/csab:103224900 | 2 |
| csab05167 | Kaposi sarcoma-associated herpesvirus infection | 3/88 | 148/5900 | 0.3803518 | 0.7487364 | 103230198/103235789/103224900 | IKBKE/CXCL1/PIK3R3 | csab:103230198/csab:103235798/csab:103224900 | 3 |
| csab00051 | Fructose and mannose metabolism | 1/88 | 32/5900 | 0.3825522 | 0.7487364 | 103242625 | ALDOC | csab:103242625 | 1 |
| csab01200 | Carbon metabolism | 2/88 | 89/5900 | 0.3845782 | 0.7487364 | 103242625/103218486 | ALDOC/ENO2 | csab:103242625/csab:103218486 | 2 |
| csab04130 | SNARE interactions in vesicular transport | 1/88 | 33/5900 | 0.3918119 | 0.7561282 | 103246629 | STX1A | csab:103246629 | 1 |
| csab04144 | Endocytosis | 4/88 | 214/5900 | 0.3967041 | 0.7589123 | 103246479/103233004/103223677/103239806 | FGFR3/EHD1/HSPA6/DNM1 | csab:103246479/csab:103233004/csab:103223677/csab:103239806 | 4 |
| csab04151 | PI3K-Akt signaling pathway | 5/88 | 280/5900 | 0.4065550 | 0.7675186 | 103237005/103245773/103246479/103224900/103223920 | IL7/SGK2/FGFR3/PIK3R3/IL6R | csab:103237005/csab:103245773/csab:103246479/csab:103224900/csab:103223920 | 5 |
| csab05165 | Human papillomavirus infection | 5/88 | 281/5900 | 0.4095532 | 0.7675186 | 103223483/103230198/103224207/103224900/103239621 | WNT7B/IKBKE/WNT2B/PIK3R3/NOTCH1 | csab:103223483/csab:103230198/csab:103224207/csab:103224900/csab:103239621 | 5 |
| csab05170 | Human immunodeficiency virus 1 infection | 3/88 | 157/5900 | 0.4168271 | 0.7675186 | 103225587/103224900/103221664 | TNFRSF1B/PIK3R3/LOC103221664 | csab:103225587/csab:103224900/csab:103221664 | 3 |
| csab00982 | Drug metabolism - cytochrome P450 | 1/88 | 36/5900 | 0.4187749 | 0.7675186 | 103230592 | FMO4 | csab:103230592 | 1 |
| csab04913 | Ovarian steroidogenesis | 1/88 | 36/5900 | 0.4187749 | 0.7675186 | 103235528 | PLA2G4C | csab:103235528 | 1 |
| csab03050 | Proteasome | 1/88 | 37/5900 | 0.4274972 | 0.7675186 | 103233253 | PSMB10 | csab:103233253 | 1 |
| csab00380 | Tryptophan metabolism | 1/88 | 38/5900 | 0.4360901 | 0.7675186 | 103232876 | LOC103232876 | csab:103232876 | 1 |
| csab04961 | Endocrine and other factor-regulated calcium reabsorption | 1/88 | 38/5900 | 0.4360901 | 0.7675186 | 103239806 | DNM1 | csab:103239806 | 1 |
| csab05204 | Chemical carcinogenesis - DNA adducts | 1/88 | 38/5900 | 0.4360901 | 0.7675186 | 103230154 | HSD11B1 | csab:103230154 | 1 |
| csab05219 | Bladder cancer | 1/88 | 38/5900 | 0.4360901 | 0.7675186 | 103246479 | FGFR3 | csab:103246479 | 1 |
| csab00140 | Steroid hormone biosynthesis | 1/88 | 39/5900 | 0.4445555 | 0.7762080 | 103230154 | HSD11B1 | csab:103230154 | 1 |
| csab04915 | Estrogen signaling pathway | 2/88 | 103/5900 | 0.4571019 | 0.7904310 | 103224900/103223677 | PIK3R3/HSPA6 | csab:103224900/csab:103223677 | 2 |
| csab04930 | Type II diabetes mellitus | 1/88 | 41/5900 | 0.4611111 | 0.7904310 | 103224900 | PIK3R3 | csab:103224900 | 1 |
| csab04217 | Necroptosis | 2/88 | 105/5900 | 0.4670729 | 0.7904310 | 103241278/103235528 | IL1B/PLA2G4C | csab:103241278/csab:103235528 | 2 |
| csab04611 | Platelet activation | 2/88 | 105/5900 | 0.4670729 | 0.7904310 | 103235528/103224900 | PLA2G4C/PIK3R3 | csab:103235528/csab:103224900 | 2 |
| csab05321 | Inflammatory bowel disease | 1/88 | 43/5900 | 0.4771788 | 0.8013689 | 103241278 | IL1B | csab:103241278 | 1 |
| csab04742 | Taste transduction | 1/88 | 44/5900 | 0.4850340 | 0.8034182 | 103229709 | SCNN1B | csab:103229709 | 1 |
| csab05022 | Pathways of neurodegeneration - multiple diseases | 6/88 | 373/5900 | 0.4857028 | 0.8034182 | 103241278/103223483/103225587/103224207/103246629/103227647 | IL1B/WNT7B/TNFRSF1B/WNT2B/STX1A/UBA7 | csab:103241278/csab:103223483/csab:103225587/csab:103224207/csab:103246629/csab:103227647 | 6 |
| csab00280 | Valine, leucine and isoleucine degradation | 1/88 | 46/5900 | 0.5003961 | 0.8094644 | 103227954 | ABAT | csab:103227954 | 1 |
| csab00510 | N-Glycan biosynthesis | 1/88 | 46/5900 | 0.5003961 | 0.8094644 | 103242981 | MGAT5B | csab:103242981 | 1 |
| csab04730 | Long-term depression | 1/88 | 46/5900 | 0.5003961 | 0.8094644 | 103235528 | PLA2G4C | csab:103235528 | 1 |
| csab00561 | Glycerolipid metabolism | 1/88 | 47/5900 | 0.5079064 | 0.8155907 | 103224742 | PLPP3 | csab:103224742 | 1 |
| csab04923 | Regulation of lipolysis in adipocytes | 1/88 | 48/5900 | 0.5153051 | 0.8155907 | 103224900 | PIK3R3 | csab:103224900 | 1 |
| csab05031 | Amphetamine addiction | 1/88 | 48/5900 | 0.5153051 | 0.8155907 | 103246629 | STX1A | csab:103246629 | 1 |
| csab04613 | Neutrophil extracellular trap formation | 2/88 | 118/5900 | 0.5291839 | 0.8315746 | 103223257/103224900 | NCF4/PIK3R3 | csab:103223257/csab:103224900 | 2 |
| csab04330 | Notch signaling pathway | 1/88 | 51/5900 | 0.5368475 | 0.8376343 | 103239621 | NOTCH1 | csab:103239621 | 1 |
| csab04622 | RIG-I-like receptor signaling pathway | 1/88 | 52/5900 | 0.5438157 | 0.8425314 | 103230198 | IKBKE | csab:103230198 | 1 |
| csab04929 | GnRH secretion | 1/88 | 53/5900 | 0.5506803 | 0.8452964 | 103224900 | PIK3R3 | csab:103224900 | 1 |
| csab05213 | Endometrial cancer | 1/88 | 55/5900 | 0.5641046 | 0.8452964 | 103224900 | PIK3R3 | csab:103224900 | 1 |
| csab04934 | Cushing syndrome | 2/88 | 127/5900 | 0.5692743 | 0.8452964 | 103223483/103224207 | WNT7B/WNT2B | csab:103223483/csab:103224207 | 2 |
| csab04918 | Thyroid hormone synthesis | 1/88 | 56/5900 | 0.5706673 | 0.8452964 | 103237469 | TG | csab:103237469 | 1 |
| csab04920 | Adipocytokine signaling pathway | 1/88 | 57/5900 | 0.5771323 | 0.8452964 | 103225587 | TNFRSF1B | csab:103225587 | 1 |
| csab05221 | Acute myeloid leukemia | 1/88 | 57/5900 | 0.5771323 | 0.8452964 | 103224900 | PIK3R3 | csab:103224900 | 1 |
| csab05152 | Tuberculosis | 2/88 | 129/5900 | 0.5778480 | 0.8452964 | 103241278/103231401 | IL1B/ITGAX | csab:103241278/csab:103231401 | 2 |
| csab05218 | Melanoma | 1/88 | 58/5900 | 0.5835010 | 0.8452964 | 103224900 | PIK3R3 | csab:103224900 | 1 |
| csab04917 | Prolactin signaling pathway | 1/88 | 60/5900 | 0.5959553 | 0.8452964 | 103224900 | PIK3R3 | csab:103224900 | 1 |
| csab04218 | Cellular senescence | 2/88 | 134/5900 | 0.5987432 | 0.8452964 | 103230197/103224900 | RASSF5/PIK3R3 | csab:103230197/csab:103224900 | 2 |
| csab01524 | Platinum drug resistance | 1/88 | 61/5900 | 0.6020436 | 0.8452964 | 103224900 | PIK3R3 | csab:103224900 | 1 |
| csab04115 | p53 signaling pathway | 1/88 | 61/5900 | 0.6020436 | 0.8452964 | 103236967 | CD82 | csab:103236967 | 1 |
| csab04260 | Cardiac muscle contraction | 1/88 | 61/5900 | 0.6020436 | 0.8452964 | 103219220 | TPM2 | csab:103219220 | 1 |
| csab05214 | Glioma | 1/88 | 62/5900 | 0.6080412 | 0.8452964 | 103224900 | PIK3R3 | csab:103224900 | 1 |
| csab05322 | Systemic lupus erythematosus | 1/88 | 63/5900 | 0.6139495 | 0.8452964 | 103221732 | LOC103221732 | csab:103221732 | 1 |
| csab05161 | Hepatitis B | 2/88 | 138/5900 | 0.6149032 | 0.8452964 | 103230198/103224900 | IKBKE/PIK3R3 | csab:103230198/csab:103224900 | 2 |
| csab04390 | Hippo signaling pathway | 2/88 | 142/5900 | 0.6305690 | 0.8452964 | 103223483/103224207 | WNT7B/WNT2B | csab:103223483/csab:103224207 | 2 |
| csab03008 | Ribosome biogenesis in eukaryotes | 1/88 | 66/5900 | 0.6311510 | 0.8452964 | 103232396 | NXF3 | csab:103232396 | 1 |
| csab04146 | Peroxisome | 1/88 | 66/5900 | 0.6311510 | 0.8452964 | 103240818 | SOD2 | csab:103240818 | 1 |
| csab04740 | Olfactory transduction | 1/88 | 66/5900 | 0.6311510 | 0.8452964 | 103218440 | ANO2 | csab:103218440 | 1 |
| csab04520 | Adherens junction | 1/88 | 67/5900 | 0.6367147 | 0.8452964 | 103218491 | PTPN6 | csab:103218491 | 1 |
| csab04727 | GABAergic synapse | 1/88 | 67/5900 | 0.6367147 | 0.8452964 | 103227954 | ABAT | csab:103227954 | 1 |
| csab05207 | Chemical carcinogenesis - receptor activation | 2/88 | 146/5900 | 0.6457427 | 0.8452964 | 103230198/103224900 | IKBKE/PIK3R3 | csab:103230198/csab:103224900 | 2 |
| csab03018 | RNA degradation | 1/88 | 69/5900 | 0.6475944 | 0.8452964 | 103218486 | ENO2 | csab:103218486 | 1 |
| csab04658 | Th1 and Th2 cell differentiation | 1/88 | 69/5900 | 0.6475944 | 0.8452964 | 103239621 | NOTCH1 | csab:103239621 | 1 |
| csab01232 | Nucleotide metabolism | 1/88 | 70/5900 | 0.6529129 | 0.8452964 | 103240330 | AMPD3 | csab:103240330 | 1 |
| csab05202 | Transcriptional misregulation in cancer | 2/88 | 148/5900 | 0.6531460 | 0.8452964 | 103215685/103218917 | PLAT/TRAF1 | csab:103215685/csab:103218917 | 2 |
| csab05220 | Chronic myeloid leukemia | 1/88 | 71/5900 | 0.6581519 | 0.8452964 | 103224900 | PIK3R3 | csab:103224900 | 1 |
| csab05010 | Alzheimer disease | 4/88 | 302/5900 | 0.6669939 | 0.8452964 | 103241278/103223483/103224207/103224900 | IL1B/WNT7B/WNT2B/PIK3R3 | csab:103241278/csab:103223483/csab:103224207/csab:103224900 | 4 |
| csab04912 | GnRH signaling pathway | 1/88 | 73/5900 | 0.6683966 | 0.8452964 | 103235528 | PLA2G4C | csab:103235528 | 1 |
| csab05212 | Pancreatic cancer | 1/88 | 73/5900 | 0.6683966 | 0.8452964 | 103224900 | PIK3R3 | csab:103224900 | 1 |
| csab05410 | Hypertrophic cardiomyopathy | 1/88 | 74/5900 | 0.6734045 | 0.8452964 | 103219220 | TPM2 | csab:103219220 | 1 |
| csab05203 | Viral carcinogenesis | 2/88 | 155/5900 | 0.6781038 | 0.8452964 | 103218917/103224900 | TRAF1/PIK3R3 | csab:103218917/csab:103224900 | 2 |
| csab04974 | Protein digestion and absorption | 1/88 | 75/5900 | 0.6783376 | 0.8452964 | 103242775 | COL20A1 | csab:103242775 | 1 |
| csab05414 | Dilated cardiomyopathy | 1/88 | 76/5900 | 0.6831971 | 0.8452964 | 103219220 | TPM2 | csab:103219220 | 1 |
| csab03015 | mRNA surveillance pathway | 1/88 | 79/5900 | 0.6973441 | 0.8452964 | 103232396 | NXF3 | csab:103232396 | 1 |
| csab04012 | ErbB signaling pathway | 1/88 | 79/5900 | 0.6973441 | 0.8452964 | 103224900 | PIK3R3 | csab:103224900 | 1 |
| csab04024 | cAMP signaling pathway | 2/88 | 161/5900 | 0.6983334 | 0.8452964 | 103223064/103224900 | ADORA2A/PIK3R3 | csab:103223064/csab:103224900 | 2 |
| csab04914 | Progesterone-mediated oocyte maturation | 1/88 | 80/5900 | 0.7019196 | 0.8452964 | 103224900 | PIK3R3 | csab:103224900 | 1 |
| csab04512 | ECM-receptor interaction | 1/88 | 81/5900 | 0.7064267 | 0.8452964 | 103220724 | SDC1 | csab:103220724 | 1 |
| csab05210 | Colorectal cancer | 1/88 | 81/5900 | 0.7064267 | 0.8452964 | 103224900 | PIK3R3 | csab:103224900 | 1 |
| csab05016 | Huntington disease | 3/88 | 242/5900 | 0.7069752 | 0.8452964 | 103240818/103246306/103246629 | SOD2/TGM2/STX1A | csab:103240818/csab:103246306/csab:103246629 | 3 |
| csab04070 | Phosphatidylinositol signaling system | 1/88 | 83/5900 | 0.7152396 | 0.8505552 | 103224900 | PIK3R3 | csab:103224900 | 1 |
| csab04726 | Serotonergic synapse | 1/88 | 84/5900 | 0.7195475 | 0.8510777 | 103235528 | PLA2G4C | csab:103235528 | 1 |
| csab05415 | Diabetic cardiomyopathy | 2/88 | 169/5900 | 0.7236808 | 0.8513892 | 103223257/103224900 | NCF4/PIK3R3 | csab:103223257/csab:103224900 | 2 |
| csab04724 | Glutamatergic synapse | 1/88 | 87/5900 | 0.7320883 | 0.8529409 | 103235528 | PLA2G4C | csab:103235528 | 1 |
| csab03013 | Nucleocytoplasmic transport | 1/88 | 89/5900 | 0.7401391 | 0.8529409 | 103232396 | NXF3 | csab:103232396 | 1 |
| csab04725 | Cholinergic synapse | 1/88 | 89/5900 | 0.7401391 | 0.8529409 | 103224900 | PIK3R3 | csab:103224900 | 1 |
| csab05145 | Toxoplasmosis | 1/88 | 90/5900 | 0.7440744 | 0.8529409 | 103223677 | HSPA6 | csab:103223677 | 1 |
| csab04020 | Calcium signaling pathway | 2/88 | 176/5900 | 0.7443848 | 0.8529409 | 103246479/103223064 | FGFR3/ADORA2A | csab:103246479/csab:103223064 | 2 |
| csab05208 | Chemical carcinogenesis - reactive oxygen species | 2/88 | 188/5900 | 0.7768280 | 0.8674803 | 103240818/103224900 | SOD2/PIK3R3 | csab:103240818/csab:103224900 | 2 |
| csab04931 | Insulin resistance | 1/88 | 99/5900 | 0.7769420 | 0.8674803 | 103224900 | PIK3R3 | csab:103224900 | 1 |
| csab04145 | Phagosome | 1/88 | 100/5900 | 0.7803257 | 0.8674803 | 103223257 | NCF4 | csab:103223257 | 1 |
| csab04071 | Sphingolipid signaling pathway | 1/88 | 102/5900 | 0.7869417 | 0.8674803 | 103224900 | PIK3R3 | csab:103224900 | 1 |
| csab04722 | Neurotrophin signaling pathway | 1/88 | 102/5900 | 0.7869417 | 0.8674803 | 103224900 | PIK3R3 | csab:103224900 | 1 |
| csab04935 | Growth hormone synthesis, secretion and action | 1/88 | 102/5900 | 0.7869417 | 0.8674803 | 103224900 | PIK3R3 | csab:103224900 | 1 |
| csab03040 | Spliceosome | 1/88 | 104/5900 | 0.7933606 | 0.8674803 | 103223677 | HSPA6 | csab:103223677 | 1 |
| csab00230 | Purine metabolism | 1/88 | 106/5900 | 0.7995883 | 0.8674803 | 103240330 | AMPD3 | csab:103240330 | 1 |
| csab04514 | Cell adhesion molecules | 1/88 | 106/5900 | 0.7995883 | 0.8674803 | 103220724 | SDC1 | csab:103220724 | 1 |
| csab05012 | Parkinson disease | 2/88 | 198/5900 | 0.8010942 | 0.8674803 | 103223064/103227647 | ADORA2A/UBA7 | csab:103223064/csab:103227647 | 2 |
| csab04152 | AMPK signaling pathway | 1/88 | 107/5900 | 0.8026322 | 0.8674803 | 103224900 | PIK3R3 | csab:103224900 | 1 |
| csab04910 | Insulin signaling pathway | 1/88 | 111/5900 | 0.8143573 | 0.8674803 | 103224900 | PIK3R3 | csab:103224900 | 1 |
| csab04926 | Relaxin signaling pathway | 1/88 | 111/5900 | 0.8143573 | 0.8674803 | 103224900 | PIK3R3 | csab:103224900 | 1 |
| csab04371 | Apelin signaling pathway | 1/88 | 113/5900 | 0.8199589 | 0.8674803 | 103215685 | PLAT | csab:103215685 | 1 |
| csab05017 | Spinocerebellar ataxia | 1/88 | 114/5900 | 0.8226967 | 0.8674803 | 103224900 | PIK3R3 | csab:103224900 | 1 |
| csab04261 | Adrenergic signaling in cardiomyocytes | 1/88 | 115/5900 | 0.8253933 | 0.8674803 | 103219220 | TPM2 | csab:103219220 | 1 |
| csab04080 | Neuroactive ligand-receptor interaction | 2/88 | 210/5900 | 0.8271430 | 0.8674803 | 103240389/103223064 | ADM/ADORA2A | csab:103240389/csab:103223064 | 2 |
| csab05034 | Alcoholism | 1/88 | 116/5900 | 0.8280494 | 0.8674803 | 103223064 | ADORA2A | csab:103223064 | 1 |
| csab01240 | Biosynthesis of cofactors | 1/88 | 121/5900 | 0.8407422 | 0.8749850 | 103225584 | DHRS3 | csab:103225584 | 1 |
| csab04921 | Oxytocin signaling pathway | 1/88 | 122/5900 | 0.8431673 | 0.8749850 | 103235528 | PLA2G4C | csab:103235528 | 1 |
| csab04140 | Autophagy - animal | 1/88 | 126/5900 | 0.8525080 | 0.8805247 | 103224900 | PIK3R3 | csab:103224900 | 1 |
| csab04120 | Ubiquitin mediated proteolysis | 1/88 | 130/5900 | 0.8612983 | 0.8854468 | 103227647 | UBA7 | csab:103227647 | 1 |
| csab04141 | Protein processing in endoplasmic reticulum | 1/88 | 147/5900 | 0.8932286 | 0.9140013 | 103223677 | HSPA6 | csab:103223677 | 1 |
| csab04360 | Axon guidance | 1/88 | 161/5900 | 0.9139748 | 0.9309002 | 103224900 | PIK3R3 | csab:103224900 | 1 |
| csab05014 | Amyotrophic lateral sclerosis | 2/88 | 284/5900 | 0.9304713 | 0.9430922 | 103225587/103232396 | TNFRSF1B/NXF3 | csab:103225587/csab:103232396 | 2 |
| csab04510 | Focal adhesion | 1/88 | 182/5900 | 0.9378490 | 0.9430922 | 103224900 | PIK3R3 | csab:103224900 | 1 |
| csab05166 | Human T-cell leukemia virus 1 infection | 1/88 | 183/5900 | 0.9388055 | 0.9430922 | 103224900 | PIK3R3 | csab:103224900 | 1 |
| csab05132 | Salmonella infection | 1/88 | 199/5900 | 0.9522714 | 0.9522714 | 103241278 | IL1B | csab:103241278 | 1 |
